# The neurological sequelae of pandemics and epidemics

**DOI:** 10.1007/s00415-020-10261-3

**Published:** 2020-10-26

**Authors:** Fernanda Valerio, Daniel P. Whitehouse, David K. Menon, Virginia F. J. Newcombe

**Affiliations:** 1 University Division of Anaesthesia, Addenbrooke’s Hospital, University of Cambridge, Box 93, Hills Road, Cambridge, CB2 0QQ UK; 2 Wolfson Brain Imaging Centre, University of Cambridge, Cambridge, UK

**Keywords:** CNS infections, Peripheral neuropathies, Pandemics/history, Meningitis, Encephalitis

## Abstract

Neurological manifestations in pandemics frequently cause short and long-term consequences which are frequently overlooked. Despite advances in the treatment of infectious diseases, nervous system involvement remains a challenge, with limited treatments often available. The under-recognition of neurological manifestations may lead to an increase in the burden of acute disease as well as secondary complications with long-term consequences. Nervous system infection or dysfunction during pandemics is common and its enduring consequences, especially among vulnerable populations, are frequently forgotten. An improved understanding the possible mechanisms of neurological damage during epidemics, and increased recognition of the possible manifestations is fundamental to bring insights when dealing with future outbreaks. To reverse this gap in knowledge, we reviewed all the pandemics, large and important epidemics of human history in which neurological manifestations are evident, and described the possible physiological processes that leads to the adverse sequelae caused or triggered by those pathogens.

## Introduction

Pandemics are large-scale outbreaks of infectious disease that can cause an excess in morbidity and mortality globally, or at least over a wide geographic area, and lead to socio-economic disruption. Increase in global travel, urbanisation, climate change, environmental degradation, displacement and consumption of wild animals are factors thought to have increased the likelihood of pandemics during the past century [79]. The majority of pathogens responsible for outbreaks can cause neurologic illness, which are frequently overlooked, under-reported and under-diagnosed. Even in tertiary centres of developed countries, up to 30% of patients with a CNS infection never receive an etiological diagnosis [135], and in low resource settings lacking diagnostic tools, neurological manifestations are often poorly characterised. Aside from the associated mortality, neurological involvement of infectious disease can lead to prolonged hospital stay and significantly increase rehabilitation time and long-term care needs after discharge [135], posing a far-reaching socioeconomic burden.

As the world deals with the Sars-CoV2 pandemic, reports of neurologic manifestations have increased. Understanding neurological complications of previous pandemics, and the pathophysiological mechanisms that underlie them, are fundamental to place the current situation in perspective, and help address the enduring consequences once current waves of acute infection subside. This narrative review assesses the neurological manifestations of past and current pandemics, to aid our understanding of the current pandemic and prepare for future outbreaks.

## Mechanisms of pathogen-mediated neurological disease

**Fig. 1:**
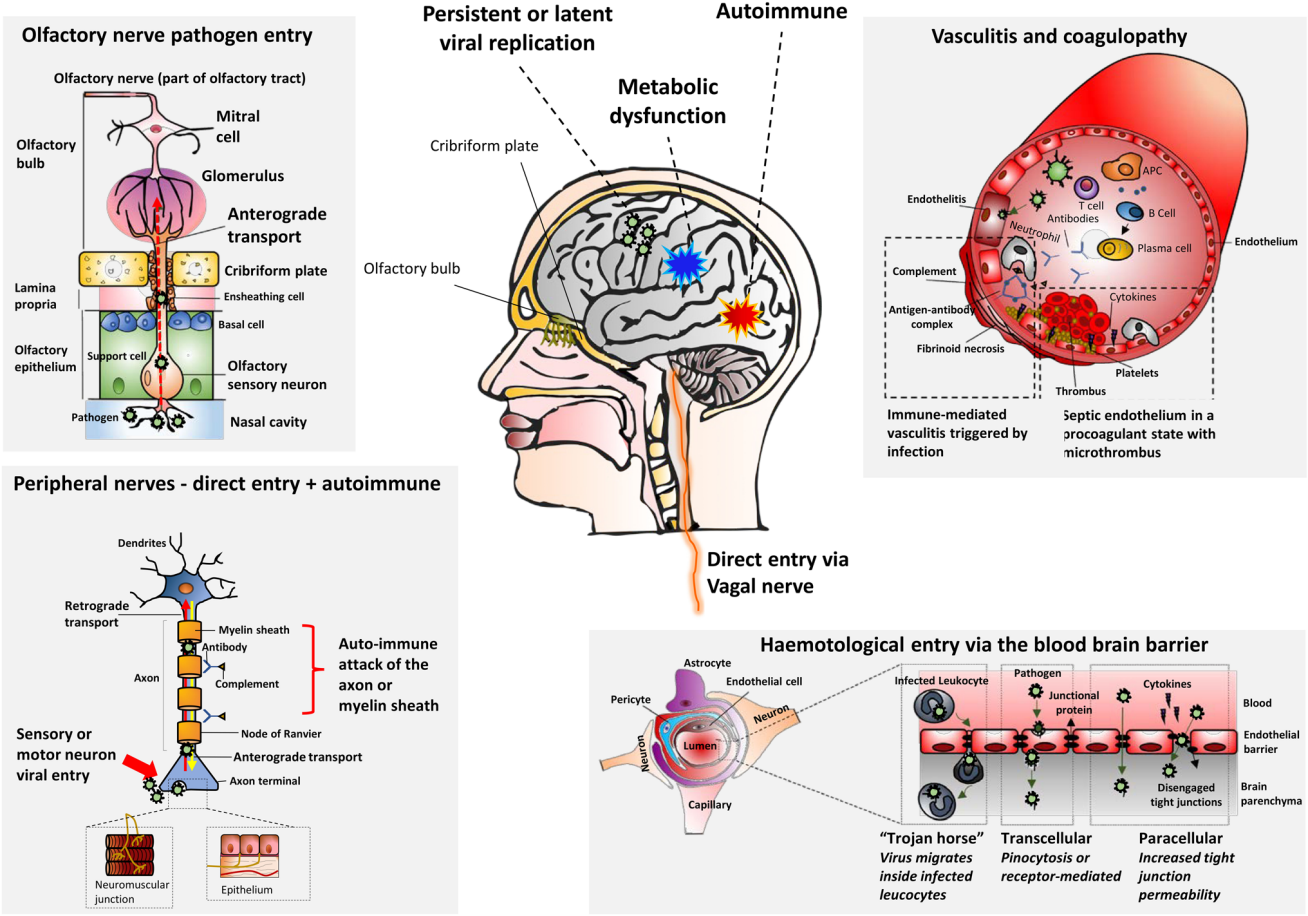
Mechanisms of injury to the nervous system

Pathogens can lead to nervous system impairment through multiple mechanisms. There may be direct infection and replication leading to the clinical syndromes of encephalitis, myelitis and meningitis [65]. Para-infectious complications such as sepsis and metabolic dysfunction related and coagulopathy can lead to encephalopathy and vascular events. The infection can also trigger an indirect immune-mediated attack both in the central and peripheral nervous system [32], as seen in Guillain–Barré syndromes (GBS) or acute disseminated encephalomyelitis (ADEM). Finally, some viruses can persist mutated or latent in the central nervous system (CNS) or peripheral ganglia, leading to potential late reactivation and clinical disease. Further details are provided in Table 1; Fig. 1.

**Table 1:** Mechanisms of pathogen-induced neurological injury

Mechanisms	Description	Neurologic manifestations
**Direct invasion**		
Blood brain barrier (BBB) (haematological entry)	Penetration of endothelial barriers via (BBB disruption associated with acute host inflammatory responses may facilitate invasion): Transcellular penetration (using pinocytosis or receptor-mediated entry) Paracellular entry (by disrupting tight junctions) Via entry of infected leukocytes from the peripheral circulation into the CNS (Trojan horse mechanism) [28, 65]	Encephalitis Meningoencephalitis Meningitis Anterior myelitis Encephalopathy
Peripheral nerves (trans-synaptic spread)	Pathogens: Move along peripheral nerves via retrograde (from axon terminal to cell body) or anterograde (from cell body to axon terminal) transport Invade the PNS by binding to receptors on axons of sensory, autonomic and motor neurons, including the olfactory and vagal nerves [68, 77, 84, 116, 142]	
**Para-infectious**		
Sepsis-associated	Diffuse disturbance of brain function as a consequence of the systemic inflammatory response of sepsis [45, 148] Presents as impaired attention and arousal [165] Release of inflammatory mediators affecting both the BBB and the cerebral microcirculation [120] (“cytokine storm”)	Encephalopathy Acute haemorrhagic leukoencephalitis (AHL)
Secondary to metabolic dysfunction	Can be isolated or in the context of sepsis and organ failure: Severe hypoxia Shock-induced hypoperfusion Metabolic disturbances Electrolyte imbalances (hyponatremia or hypernatremia, hypocalcaemia or hypercalcemia)	Seizures Encephalopathy Diffuse ischaemia
Secondary to coagulopathy and vasculitis	Hyperinflammation in SIRS and sepsis leads to coagulopathy and a prothrombotic state Both direct pathogen invasion and the proinflammatory state of sepsis lead to endothelial damage [144], which then shifts to a procoagulant state and has increased vascular permeability [57, 126] Infections may cause vasculitis, either due to pathogen invasion, exaggerated immune response, or immune dysregulation triggered by bacterial toxins or antigens [136]	Stroke Cerebral venous thrombosis Intracranial haemorrhage Peripheral neuropathy
**Post-infectious**		
Autoimmune	Triggered especially by viruses, which serve as antigenic stimulus and lead to lesions of the PNS and CNS Associated with molecular mimicry between the pathogen and molecules on the axolemmal surface, glial membranes at the node of Ranvier [150] or myelin proteins of the host, which leads to T-cell activation and an autoimmune response [106]	Guillain-Barré syndromes Acute disseminated encephalomyelitis (ADEM) Transverse myelitis Acute motor axonal neuropathy (AMAN) Acute inflammatory demyelinating polyneuropathy (AIDP)
Persistence and latency of viral infections	After direct invasion of the nervous system Persistent viral infections: continuous viral replication Latent viral infections: dormant state with minimal or no production of viral material [84].Can be reactivated upon host immunosuppression, and the activation of both innate and adaptive immune system can disrupt CNS homeostasis [65]	Neurocognitive disorders (HIV) Subacute sclerosing panencephalitis (measles)

*BBB* blood–brain-barrier, *PNS* peripheral nervous system, *CNS* central nervous system, *AHL* acute haemorrhagic leukoencephalitis, *ADEM* acute disseminated encephalomyelitis, *HIV* human immunodeficiency virus

## Pathogens responsible for pandemics and important epidemics throughout history

**Fig. 2:**
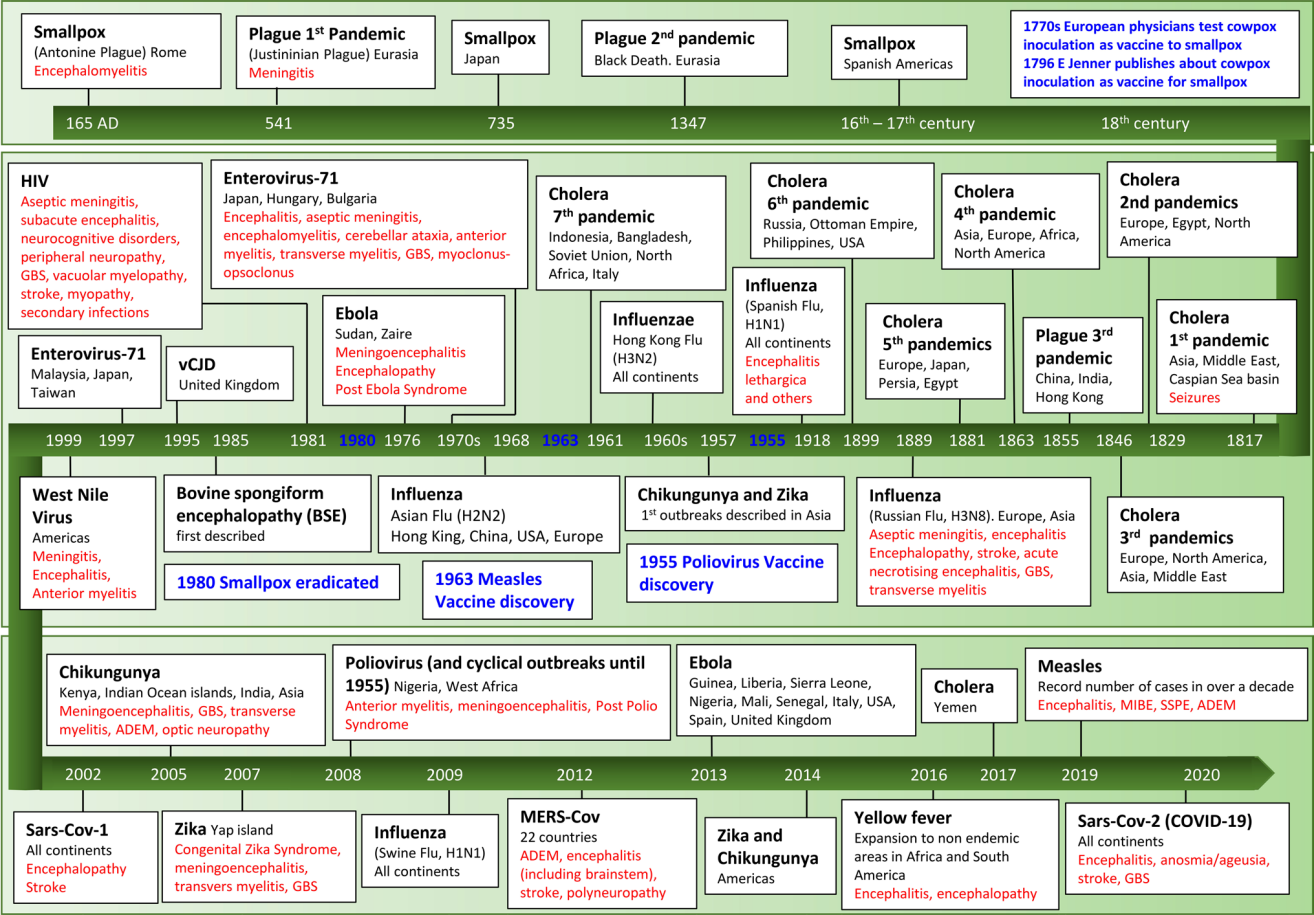
Timeline of important pandemics including key neurological complications. These are noted for the first pandemic caused by an infection

Pathogens are divided according to the main mode of transmission (vector-borne, water/food-borne and direct or indirect with infected individuals). Its features, including notable pandemics, non-neurological and neurological manifestations may be found in Fig. 2 and Table 2. We have summarised imaging and laboratory findings in Table 3. A more complete description of the neurological manifestations follows.

**Table 2:** Pandemics and selected epidemics in which neurological manifestations were reported

Disease (pathogen)	Event/distribution	Mode of transmission	Incubation period	Non-neurological features	Neurologic manifestations	Mechanism of neurological injury	Treatment
Bubonic plague (Yersinia pestis)	541: Justinian plague—Eurasia 1347: Bubonic plague—Eurasia 1855: Third world plague—China, India, Hong Kong Current: Seasonal epidemics in Africa (mainly Madagascar)	Bite of infected rodents’ fleas Direct contact with infected bodily fluids, fomites, and inhalation of infected respiratory droplets	1–7 days	Initial symptoms are fever, malaise, myalgia. Lymphadenitis develops near the location of flea bite (bubo), characterizing the bubonic form, (most common). Other forms are the septicaemic and pulmonary plague	Meningitis (may present late in the course of disease even in patients under antibiotics)	Direct invasion	Antibiotic therapy with chloramphenicol if plague meningitis. For other forms, gentamicin OR doxycycline OR ciprofloxacin can be used
West Nile (West Nile virus)	Late 1990s: Romania, Russia, Israel 1999–2003: Americas	Bite by virus-carrying *Culex* mosquitoes. Birds are reservoirs in nature	3–14 days	Most infected are asymptomatic. Between 20 and 40% will develop a flu-like illness with fever, headache, malaise, myalgia, skin rash and gastrointestinal symptoms	Meningitis Encephalitis Anterior myelitis	Direct invasion	Supportive care. No controlled trial has successfully tested the efficacy of specific drugs
Japanese Encephalitis (Japanese encephalitis virus)*	Endemic in 24 countries of tropical South East Asia and West Pacific Large epidemics every 2–15 years in temperate areas of Asia	Bite by virus-carrying *Culex* mosquitoes. The virus exists in an enzootic cycle between birds and mosquitoes with pigs as amplifying hosts and humans as dead-end hosts	5–15 days	Vast majority of infections are either asymptomatic or very mild. If present, symptoms include self-limiting fever and eventual coryza and/or diarrhoea	Encephalitis Anterior myelitis GBS Transverse myelitis ADEM Associated with NMDA encephalitis	Direct invasion Post-infectious—autoimmune	Supportive care during the acute phase. Post infectious autoimmune conditions receive the standard immunomodulatory treatment for the condition
Zika (Zika virus)	2007: Yap island (western Pacific Ocean) 2013: French Polynesia 2014–2016: Americas 2016: Singapore, Vietnam, Thailand, Guinea-Bissau, Angola 2018: India	Bite of virus-carrying *Aedes* mosquitoes, sexual relations and blood transfusion. Vertically transmitted during pregnancy	3–14 days	Infected adults are either asymptomatic (50–80%) or have mild flu-like symptoms: fever, rash, arthralgia, conjunctivitis and myalgia). Maternal-foetal transmission occurs in 20–30% of cases, among which 4–7% will lead to foetal loss	Congenital Zika syndrome: microcephaly, subcortical calcifications, corpus callosum, cortex malformations, retinal alterations Meningoencephalitis Transverse myelitis GBS	Direct invasion Post-infectious—autoimmune	Supportive care during the acute phase. Post infectious autoimmune conditions receive the standard immunomodulatory treatment for the condition
Chikungunya fever (Chikungunya virus)	1960s: Asia 2005–2007: Kenya, Indian Ocean islands, India, South East Asia 2007: Italy 2014: France 2014–current: Americas 2017–current: Pakistan	Bites of virus-carrying mosquitoes, predominantly *Aedes aegypti* and colder-climate tolerant *Aedes albopictus.* Other forms include: blood borne transmission, vertical transmission during 2nd trimester, intrapartum transmission when mother is viraemic during delivery	3–7 days	Fever, polyarthralgia (usually bilateral and symmetric), headache, myalgia, conjunctivitis, arthritis, nausea, vomiting and maculopapular rash, for 7–10 days. Some patients persist with arthralgia for months to years after the acute disease	Acute encephalitis Meningoencephalitis GBS (including Miller Fisher and Bickerstaff’s syndromes) Transverse myelitis Myeloradiculitis ADEM Optic neuropathy	Direct invasion Post-infectious—autoimmune	Supportive care during the acute phase. Post infectious autoimmune conditions receive the standard immunomodulatory treatment for the condition
Malaria (*Plasmodium falciparum*)**	Endemic with local outbreaks in 91 tropical and subtropical countries of Africa, Central and South America and Asia (nearly half of the world population). Eliminated from Europe in 1975 Epidemic potential expected to increase with increasing temperatures associated with climate changes	Mosquito bite of infective female *Anopheles.* Also, though blood transfusion, organ transplant, shared use of needles, and vertically during pregnancy	9–14 days	Fever, headache, sweats, chills, malaise, myalgia, gastrointestinal symptoms The classical (but seldom observed) malaria attack lasts 6–10 h, consisting of a cold stage (shivering), a hot stage (fever, headaches, vomiting) and a sweating stage (sweats and fatigue)	Encephalopathy Seizures	Para-infectious- secondary to sequestration of erythrocytes into cerebral blood vessels, release of cytokines, BBB permeability and metabolic dysfunction	Supportive care during the acute phase including correction of metabolic dysfunction
Yellow fever (yellow fever virus)*	Endemic in 47 countries of Africa, South and Central Americas nineteenth century: USA, Europe (Atlantic ports), the Caribbean, Central America 2016–current: expansion to non-endemic areas in Africa and South America	Mosquito bites from *Aedes* and *Haemogogus* species. Monkeys are also infected and are reservoirs in jungle areas	3–6 days	Fever, myalgia, chills, backache, headache, lack of appetite, nausea and vomiting for 3–4 days. Some are asymptomatic. Between 15 and 25% of infected enter a 2nd toxic phase with fever, vomiting, epigastric pain, renal failure, haemorrhagic diathesis, transaminase and direct bilirubin rise with deep jaundice	Febrile seizures in the acute phase (young children) Encephalitis Encephalopathy Yellow fever vaccine associated neurotropic disease: Meningitis, encephalitis, myelitis, GBS and ADEM	Direct invasion Post-infectious—autoimmune (associated with the yellow fever 17D vaccine) Para-infectious—secondary to metabolic dysfunction	Supportive care during the acute phase, including correction of metabolic dysfunction Post infectious autoimmune conditions receive the standard immunomodulatory treatment for the condition
Dengue (dengue virus)*	Evolved from sporadic cases in 1970s to endemic in over 100 countries worldwide with explosive outbreaks in new areas	Bite of virus-carrying mosquitoes, predominantly *Aedes aegypti* and colder-climate tolerant *Aedes albopictus*. Perinatal transmission occurs when the mother is infected near delivery. Breast milk and bloodborne transmission are also possible	4–10 days	40–80% are asymptomatic. Self-limiting symptoms last 5–7 days and include high fever, headache (specially retroorbital), myalgia, arthralgia and, rash. Around 5% will have the severe form of disease, manifest during the defervescence period (after the 1st week) in which there is plasma leakage due to increased vascular permeability, with or without bleeding and can lead to shock and severe organ involvement	Encephalopathy Encephalitis Aseptic meningitis ADEM Transverse myelitis GBS Mononeuropathies of cranial nerves Optic neuropathy Muscle dysfunction Intracranial haemorrhages	Direct invasion Para-infectious—secondary to metabolic dysfunction ? Para-infectious—secondary to coagulopathy and vasculitis Post-infectious—autoimmune	Supportive care during the acute phase and for para infectious manifestations. Post infectious autoimmune conditions receive the standard immunomodulatory treatment for the condition
Poliomyelitis (Poliovirus)	End of nineteenth century—1955: Large cyclical outbreaks during summer on Northern Europe and United States 2008: Nigeria and West Africa	Oral-faecal route by ingestion of contaminated water or food. The virus multiplies in the oropharyngeal and intestinal mucosa from where it spreads for target organs, including the CNS	7–10 days	Most infections are either asymptomatic or accompanied by mild flu-like symptoms like fever fatigue, headache and vomiting	Anterior myelitis Meningitis Encephalitis Post-polio syndrome	Direct invasion	Supportive care. Rehabilitation in the chronic phase
Enterovirus-71 (Enterovirus-71)*	Cyclical epidemics in the Asia–Pacific region every 2–3 years 1970s: Japan, Bulgaria and Hungary 1980s: Hong Kong, Australia 1997–1998: Malaysia, Japan, Taiwan 2008—China	Oral-faecal route by ingestion of contaminated water or food. The virus multiplies in the oropharyngeal and intestinal mucosa from where it spreads for target organs, including the CNS	3–10 days	In children, it can cause hand-feet-mouth disease: childhood exanthema with fever, papulovesicular rash on the palms and soles and oral ulcers, manifesting also as upper respiratory tract infection and gastroenteritis. Adults are frequently asymptomatic	Encephalitis, mainly brainstem Aseptic meningitis Encephalomyelitis Cerebellar ataxia Anterior myelitis Transverse myelitis GBS Myoclonus-opsoclonus	Direct invasion Post-infectious—autoimmune	Supportive care during the acute phase. Post infectious autoimmune conditions receive the standard immunomodulatory treatment for the condition
Variant Creutzfeldt-Jakob disease (bovine spongiform encephalopathy [BSE] prion)	1985: Bovine spongiform encephalopathy (BSE) described 1995: Beginning in the UK and spread to 13 countries	Ingestion of contaminated food (especially bovine meat), and rarely via blood transfusion or organ transplantation	15–20 years	Symptoms are eminently associated with the CNS infection	Psychiatric manifestations: depression, delusions, hallucinations Sensory disturbances, especially pain Dementia Ataxia Movement disorders: myoclonus, chorea, tremor	Direct invasion	Symptomatic management of neuropsychiatric disturbances. Palliative care during the inexorable last stages of the disease
Cholera *(Vibrio cholerae)*	1817–1824: 1st Cholera pandemic—Asia, Middle East, Caspian Sea basin 1829–1837: 2nd Cholera pandemic—Europe, Egypt, North America 1846–1860: 3rd Cholera pandemic—Europe, North America, Asia, Middle East 1863–1875: 4th Cholera pandemic—Asia, Europe, Africa, North America 1881–1896: 5th Cholera pandemic—Europe, Japan, Persia, Egypt 1899–1923: 6th Cholera pandemic—Russia, Ottoman Empire, Philippines, USA 1961–1975: 7th Cholera pandemic—Indonesia, Bangladesh, Soviet Union, North Africa, Italy 1991–94: South America, DRC (then Zaire) 2010—Haiti, Dominican Republic 2011–2018—Nigeria, DRC, Ghana, Sierra Leone, Ghana, Tanzania, Somalia, Algeria, Zimbabwe 2017–current: Yemen	Oral-faecal route by ingestion of contaminated food or water	12 h–5 days	Most infected are asymptomatic but still display faecal shedding of the bacteria. Symptoms can range from mild to severe and include watery diarrhoea and vomiting. Stools can resemble rice water, with flakes of mucus, with a fishy odour. There is no fever. Dehydration can rapidly ensue, potentially leading to hypovolemic shock	Seizures, secondary to electrolytic disturbances Peripheral neuropathy—reported during an outbreak in 2013	Para-infectious—secondary to metabolic dysfunction	Aggressive rehydration and electrolyte disturbances correction (both oral and intravenous), antibiotic therapy with Doxycycline (1st line for adults) or azithromycin (1st line for children and pregnant women) for the moderately and severely ill patients, zinc supplementation (among children)
Flu (influenza virus)	1889: Russian Flu—started in Siberia and Kazakhstan (H3N8), spread to Europe and Asia 1918–1920: Spanish Flu (H1N1)—first observed in Europe and USA before going global 1957: Asian Flu (H2N2)—start in Hong Kong, spread to China, USA and Europe 1968: Hong Kong flu (H3N2)—global 2009: Swine flu (H1N1 pdm09)—global	Direct contact with respiratory droplets from infected people or fomites	1–4 days	Common symptoms include fever, chills, cough, sore throat, runny nose, myalgia, headache, fatigue, gastrointestinal symptoms (more frequent in children). Complications include pneumonia, myocarditis and sepsis. Pregnant women, those aged < 5 or > 65 and who have underlying health conditions are at increased risk of complications	Aseptic meningitis Encephalitis Encephalopathy Stroke Acute necrotising encephalitis Kleine-Levin syndrome GBS Transverse myelitis Encephalopathic condition of Reye’s syndrome Encephalitis lethargica Post-encephalitic Parkinsonism	? Direct invasion Para-infectious—septic-related Para-infectious—secondary to metabolic dysfunction Para-infectious—secondary to coagulopathy Post-infectious—autoimmune	Supportive care. Both oseltamivir and high dose corticosteroids can be used in the acute phase. Post infectious autoimmune conditions receive the standard immunomodulatory treatment for the condition
Coronaviruses (Sars-CoV, MERS-CoV, Sars-Cov2)	2002–03: Sars-CoV—global (30 countries), start in China 2012: MERS-CoV 22 countries, start in the middle East 2019: Sars-Cov2—global	Direct contact with infected people’s respiratory droplets or fomites	2–14 days	Some are asymptomatic. For these coronaviruses most will develop mild upper respiratory tract symptoms such as cough, sore throat, coryza, fever, chills, fatigue, myalgia, headache nausea. A parcel will evolve with severe pneumonia and respiratory failure	Encephalopathy Meningoencephalitis Acute necrotising encephalitis Optic neuritis Stroke Myopathy GBS (including Miller Fisher and Bickerstaff’s syndromes) Mononeuropathies of cranial nerves ADEM	Direct invasion Para-infectious—septic-related Para-infectious—secondary to metabolic dysfunction Para-infectious—secondary to coagulopathy and vasculitis Post-infectious—autoimmune	Supportive care during the acute phase. Post infectious autoimmune conditions receive the standard immunomodulatory treatment for the condition
Ebola (Ebola virus)	1976: Sudan, DRC (then Zaire) 1995–1997: Gabon, DRC (then Zaire) 2001–2003: Uganda, Gabon, Republic of Congo 2007–2009: DRC, Uganda 2013–2016: Guinea, Liberia, Sierra Leone, Nigeria, Mali, Senegal, Italy, USA, Spain, United Kingdom 2018–current: DRC, Uganda	Contact with virus-carrying bats (main reservoir) or with infected wild animals (intermediary hosts). It easily spreads via direct contact with bodily fluids or fomites (caring for the sick and handling deceased patients is particularly high risk) Also transmitted via sexual contact (up to 12 months after the acute infection)	2–21 days	Early symptoms include high fever, malaise and body aches for 3 days, evolving to gastrointestinal symptoms which include nausea, large-volume diarrhoea and vomiting for 7—10 days. At this stage, some patients will go into shock and can present with haemorrhagic manifestations (conjunctival, gastrointestinal and mucosal bleeding)	Meningoencephalitis Encephalopathy Post Ebola syndrome: memory loss, headaches, muscles aches among survivors	Direct invasion Para-infectious—septic-related Para-infectious—secondary to metabolic dysfunction	Supportive care with early rehydration, correction of electrolyte disturbances and treating secondary infections
Measles (measles virus)*	Until 1963: massive global outbreaks every 2–3 years, until vaccine is developed, decreasing number of cases worldwide 2019–current: outbreaks in all regions of the world (increase in number of cases and resurgence in areas of Europe and USA in the last 10 years)	Direct contact with respiratory droplets and aerolised particles from infected people and fomites. Aerolised particles can remain in the air for up to 2 h	7–14 days	Starts with high fever and one or more of the three symptoms: coryza, cough and conjunctivitis. Koplik spots (small white spots) appear in the mouth 2–3 days after start of symptoms. Maculopapular rash appears 3–4 days after the onset of symptoms	Acute Encephalitis (primary measles encephalitis) Acute post-measles encephalitis Measles inclusion-body encephalitis (MIBE) Subacute sclerosing panencephalitis (SSPE) ADEM	Direct invasion Post-infectious—persistent or latent infection Post-infectious- autoimmune	Supportive care in the cute phase. Acute post measles encephalitis can be treated with corticosteroids, intravenous IgG is a possible 2nd line treatment. MIBE treatment is supportive and ribavirin is reported as beneficial. SSPE patients receive palliative care an symptoms control
AIDS (human immunodeficiency virus-HIV)	1981–current: global	Exchange of body fluids from infected people, including blood, breast milk, semen and vaginal secretions. The virus is also transmitted vertically during pregnancy and delivery	2–6 weeks for the 1st acute infection symptoms 2–15 years until the development of AIDS	The acute infection presents as a flu-like illness with fever, sore throat, body rash, fatigue, myalgia and lymphadenopathy for 1–2 weeks. After years of chronic infection symptoms related to the immune system damage appear and are related to the opportunistic infections that ensue. They include weight loss, chronic diarrhoea, fever, skin lesions, and any other symptom related to organ damage associated with the opportunistic infections	Aseptic meningitis Subacute encephalitis Neurocognitive disorders Peripheral neuropathy GBS Vacuolar myelopathy Stroke Amyotrophic lateral sclerosis-like Myopathy Predisposes secondary infections due to reactivated viruses: Progressive multifocal leukoencephalopathy (PML) (JC virus), primary CNS lymphoma (Epstein-Barr virus), neurocryptococcosis (Cryptococcus neoformans), neurotoxoplasmosis (Toxoplasma gondii)	Direct invasion Para-infectious—secondary to coagulopathy and vasculitis Post-infectious—autoimmune Post-infectious—persistent or latent infection (including secondary reactivation of other latent viruses)	Early initiation of ART against HIV is effective against both direct effects of the virus and complications secondary to opportunistic infections. It has also shown to slow progression of cognitive disorders associated with the disease Treatment of peripheral neuropathy involves both alleviation of pain and prevent progression by either initiating ART or suspending ART-related peripheral neuropathy
Smallpox (Variola virus)	Endemic in Asia since Antiquity Endemic in Europe since eleventh century with various outbreaks 165AD:Antonine Plague—Rome 735–737: Japan sixteenth-seventeenth century: Americas—decimation of native populations eighteenth century: USA	Direct contact with fluids from patients’ sores, fomites and respiratory droplets from infected people	7–19 days	After 2–4 days of fever, myalgia, headache, fatigue and eventual vomiting. An early rash on the tongue and mouth installs followed by generalised skin rash. It progresses to pustular rash around the 6th day, which then forms a crust and a scab. 3 weeks after the start of rash most scabs will have fallen off	Post-infectious encephalomyelitis Post-vaccinal encephalomyelitis	Post-infectious—autoimmune	Supportive care

Immunomodulatory treatment for post infectious complications of autoimmune nature (such as Guillain–Barré, Miller Fischer and Bickerstaff’s syndromes) usually involve intravenous immunoglobin or plasmapheresis. ADEM is typically treated with high dose intravenous corticosteroids, regardless of its precipitating cause

*DRC* Democratic Republic of Congo, *USA *United States of America, *CNS* central nervous system, *GBS* Guillain–Barré syndromes, *ADEM* acute disseminated encephalomyelitis, *Sars* severe acute respiratory syndrome, *MERS* Middle East respiratory syndrome, *ART* antiretroviral therapy, *BBB* blood brain barrier

*Where diseases are endemic around the world with outbreaks/epidemics, dates are limited

**Plasmodium vivax has rarely been reported as a cause of Encephalopathy in adults in Asia

**Table 3:** Imaging and laboratory features of each pandemic disease

Disease	Imaging	Laboratory
Plague [8, 34, 35, 82, 109, 158]	Only once case report in the literature, of paediatric plague with positive PCR in the CSF for *Y. pestis* (but negative culture), with normal MRI	CSF: moderate neutrophil pleocytosis, increased protein, low glucose. Lymphocytic pleocytosis and normal glucose have also been described in some patients Bloods: neutrophilic leucocytosis
West Nile virus [29, 123, 131, 147]	Brain CT is usually normal. If present, Brain MRI abnormalities are more likely to be seen during the 1st week and include leptomeningeal enhancement, T2/Flair hyperintense lesions in periventricular areas, deep brain structures (thalami, basal ganglia, red nucleus, cerebral peduncle, substantia nigra) and mesial temporal lobes. DWI sequences may detect lesions before T2/FLAIR Most AFP cases have normal images, but some may present with abnormalities in the anterior horn and roots	CSF: pleocytosis, which can be neutrophilic or lymphocytic, normal glucose and elevated protein in encephalitis or meningitis. Similar findings in AFP but with raised protein levels Bloods: leucocytosis, elevated AST, ALT and serum lipase are commonly described
Japanese encephalitis [131, 140, 145]	CT: bilateral thalamic hypodensities, which may be haemorrhagic and only visualised in the MRI. MRI changes include hyperintense lesions of thalamus, midbrain, pons, cerebellum, basal ganglia and cerebral cortex	CSF: usually lymphocytic pleocytosis (though it can be acellular), normal protein and glucose levels Blood: neutrophilia and hyponatremia are frequent
Zika virus [7, 90, 93, 94, 105]	Brain MRI abnormalities of symptomatic congenital Zika syndrome affects both white and grey matter and include: severe microcephaly, ventriculomegaly, skull collapse, florid grey-matter interface calcifications, corpus callosum anomalies, cerebral cortex thinning, abnormal gyral patterns, pontine atrophy, cerebellum hypoplasia, chorioretinal atrophy, microphthalmia, cataracts and optic nerve atrophy. Neuroimaging of encephalitic adult cases in non-specific and can be normal. Most commonly reported findings are asymmetric subcortical T2/Flair hyperintense lesions (also seen in DWI)	CSF: moderate pleocytosis and mildly raised protein, with mostly normal glucose in meningoencephalitis. In GBS, usually normal WBC and increased protein levels Blood: leukopenia, thrombocytopenia, increase transaminases levels
Chikungunya fever [19, 41, 42, 86, 146]	Brain CT and MRI can be normal even in encephalitic cases. Both imaging modalities can show unspecific oedema and haemorrhage in different areas of the cerebrum during the acute phase. ADEM cases present with typical confluent areas of T2/FLAIR hyperintensity consistent with demyelination. Myelopathic patients have spinal cord T2/Flair hyperintense lesions in the affected segment	CSF: pleocytosis (although it can be very modest or normal) and raised protein in meningoencephalitis and myeloradiculitis Blood: lymphopenia (almost always present), thrombocytopenia, elevated AST and ALT and hypocalcaemia
Malaria [78, 107, 111, 153]	Brain imaging findings do not correlate with parasitaemia, and can be normal. CT may display vasogenic oedema specially involving the posterior brain, ischaemic hypodensities in thalamus and cerebellar white matter. Brain MRI shows non-specific T2WI hyperintensities in the thalamus, periventricular white matter, corpus callosum, occipital sub-cortex and basal ganglia	CSF: very mild pleocytosis, low glucose and increased protein levels Blood: Anaemia, hyperbilirubinemia (due to haemolysis), thrombocytopenia, haemoglobinuria and elevated transaminases are usually present in a range of severities. Young children and pregnant women may have hypoglycaemia and metabolic acidosis
Yellow fever [85, 89]	There’s a paucity of MRI studies in yellow fever-related encephalitis. Case reports have described lesions associated with the rare complication of yellow fever 17D vaccination with unspecific T2WI hyperintense lesions in cerebral peduncles, medulla, spinal cord and cerebral white matter	There’s scarce information about CSF in yellow fever in the literature Bloods: leukopenia (with relative neutropenia), thrombocytopenia, elevated aminotransferases and bilirubin levels, increased prothrombin time (PT), albuminuria
Dengue fever [17, 49, 129]	Brain CT may reveal intraparenchymal foci of haemorrhages. Brain MRI of meningoencephalitis frequently shows hyperintensity on T2WI and DWI in both thalami, basal ganglia, cortical grey matter and subcortical white matter. Petechial haemorrhages and diffuse brain oedema are common	CSF: frequently normal in most encephalitic cases. Modest lymphocytic pleocytosis with normal or raised protein levels may be present in dengue myelitis, encephalitis or meningitis Blood: leukopenia, thrombocytopenia and rising haematocrit
Poliomyelitis [38, 58, 63, 81]]	MRI show T2W hyperintense ventral motor tracts both in the spinal cord and in the motor cortex	CSF: pleocytosis (neutrophilic in the 1st days which then progresses to lymphocytic), mildly elevated protein levels and normal glucose in the acute phase
Enterovirus-71 [59, 67, 100, 125, 130, 133]	In encephalitic patients, brainstem is the most affected site. Brain MRI shows T2W hyperintense in the midbrain, dentate nuclei, dorsal aspect of the pons (pontine tegmentum), basal ganglia and medulla. Usually there’s no supratentorial involvement	CSF: mild lymphocytic pleocytosis (though it may be normal), and usually normal protein and glucose levels in acute encephalitic patients Bloods: leucocytosis, mainly neutrophilic, especially in those with central involvement
Variant Creutzfeldt-Jakob disease [15, 88]	Brain MRI of most cases of vCJD show the characteristic “pulvinar sign”—an area of high signal in T2W in the posterior thalamus rather than any other area. Other areas which may be involved include dorsomedial thalamic nuclei and the periaqueductal grey matter. Hyperintensities in T2Wand DWI in the caudate, putamen and cortical areas, characteristically involved in sCJD, may also affected vCJD patients	CSF: usually normal, sometimes with modest total protein elevation. CSF 14-3-3 is positive only in half of vCJD patients
Influenzae [1, 18, 30, 44, 51]	Encephalitic patients may present with normal imaging. Specific encephalitic syndromes have been described in those patients, divided in splenial sign (T2 and DWI high signal in the splenium of corpus callosum); ANE-pattern (acute necrotising encephalitis- hyper signal in T2WI in thalami, midbrain, pons, cerebellum, centrum semiovale); PRES pattern (hyperintense signal in T2WI in centrum semiovale, more prominent posteriorly); malignant brain oedema (diffuse brain oedema). Post infectious cerebellitis displays T2WI hyperintense images in the cerebellum, with brainstem compression and hydrocephalus. Encephalitis lethargica patients presented with loss of neurons in the midbrain, subthalamus and hypothalamus. Post viral parkinsonism subjects had depigmentation of the substantia nigra and locus coeruleus	CSF: normal or with pleocytosis, proteins frequently elevated, normal glucose, in encephalitic cases Bloods: variable, may be closer to normal. Frequent lymphocytopenia, thrombocytopenia, elevated AST, CRP
Coronavirus [55, 66, 70, 102, 162, 163, 165]	While there’s scarce information about brain images of patients infected with Sars-Cov1 and MERS-CoV, recent studies have reported variated lesions in patients with neurologic manifestations secondary to Sars-Cov2 and novel information comes up every day. Within the pleiad of described lesions, the most frequently reported are SWI abnormalities (with ovoid or tubular shape) affecting mainly the splenium, juxta cortical U-fibres and main white matter tracts, non-confluent white matter hyperintense lesions on T2WI and DWI associated or not with haemorrhagic lesions and often involving corpus callosum and middle cerebellar peduncles, symmetric thalamic lesions with oedema, petechial haemorrhage and necrosis compatible with ANE (with variable extension to the brainstem, cerebral and cerebellar white matter tracts) Other described lesions include prominence of optic nerve subarachnoid spaces sign abnormalities in the medial temporal lobe and olfactory bulb, contrast enhancement of intraparenchymal lesions, leptomeninges and cranial nerves. Some patients also developed T2WI hyperintensities in the periventricular areas and spinal cord, suggestive of demyelination, and ADEM-compatible lesions in COVID 19 patients have been described	CSF: normal or with pleocytosis, with variable degrees of proteinorrhaquia and generally normal glucose Bloods: variable. Frequent findings are lymphopenia, decreased eosinophils, decreased albumin, raised CRP, increased LDH and increased interleukin-6
Ebola [10, 21, 22, 60, 80, 115]	Patients with presumed Ebola meningoencephalitis are rarely scanned. Case reports have described punctate T2WI hyperintense lesions in the subcortical white matter, corpus callosum and peri fourth ventricle, compatible with microvascular lesions	CSF: few case reports, evaluated only to detect viruses’ genetic material Bloods: leukopenia (earlier in the disease) or leucocytosis, thrombocytopenia, elevated transaminases (AST > ALT). Electrolyte disturbances with the disease-associated volume loss: hypo/hypernatremia, hypokalaemia, hypocalcaemia and hypomagnesemia. Coagulation abnormalities may occur
Measles [6, 16, 39, 47, 74, 124]	In the acute phase and acute post-measles encephalitis brain MRI shows multifocal hyperintense T2WI in both cerebral hemispheres, involvement of dorsal striatum and cortex oedema. MIBE presents with T2WI and DWI hyperintense lesions in the brainstem, cerebellum, multifocal cortical and subcortical grey matter lesions (including basal ganglia and thalamus), without contrast enhancement SSPE has a progressive course, initially with patchy asymmetric hyperintense lesions in T2WI in the white matter of both parietal and temporal lobes. It progresses to involve the corpus callosum and basal ganglia, culminating with generalised encephalomalacia	CSF: acute encephalitis and acute post-measles encephalitis: lymphocytic pleocytosis with high protein and mildly low glucose MIBE—CSF is usually normal, but may present with pleocytosis and elevated protein. SSPE—only high titres of measles antibody Bloods: Leukopenia, thrombocytopenia and T cell cytopenia during the acute infection
HIV [54, 95, 110, 132] [5, 24, 99, 117, 118, 127, 134, 161]	HIV-associated encephalopathy usually presents with symmetric T2WI hyperintense lesions affecting mainly periventricular and deep white matter, with associated encephalomalacia, with no mass effect or enhancement. HIV-associated cerebral vasculopathy may lead to multiple nodular and fusiform aneurysms of large and medium arteries which can cause subarachnoid or intraparenchymal haemorrhages or embolic infarcts. HIV-related vacuolar myelopathy presents with spinal cord atrophy (mainly at the thoracic level, but cervical cord may be affected) with frequent bilateral symmetric dorsal column involvement CNS opportunistic infections in AIDS patients have characteristic dependent of its agent, the most common being: - Neurotoxoplasmosis typically causes nodular lesions in the basal ganglia and at the corticomedullary junction with 1–3 cm and perilesional oedema. Brain MRI shows concentric alternating zones of hypo/hyper and isointense in T2WI with post contrast ring enhancement - CMV encephalitis shows non-specific T2WI hyperintensities in the periventricular white matter with no mass effect and no enhancement - Neurocryptococcosis usually leads to lesions with little enhancement, spread along perivascular spaces mostly in the basal ganglia and white matter of cerebral hemispheres, brainstem and cerebellum. Dilated perivascular spaces can coalesce into gelatinous pseudocysts (“soap bubble”) with high signal in T2WI. Cryptococcomas are usually T2WI hyperintense nodular lesions in the cerebral parenchyma with variable enhancement. The meningeal disease usually shows leptomeningeal and pachymeningeal enhancement - PML is typically multifocal and asymmetric with periventricular and subcortical demyelinating lesions, especially subcortical U-fibres in the parieto-occipital areas. Multiple punctate high T2 signal lesions around the involved area (“milky way sign”) differentiate it from multiple sclerosis lesions - CNS lymphoma classically presents as supratentorial mass lesions which are T1 hypointense, T2 iso/hypointense with high signal in DWI and homogeneous enhancement with sub ependymal extension and crossing of the corpus callosum	CSF: HIV aseptic meningitis: monocytic or lymphocytic pleocytosis with high or normal protein and normal glucose levels Specific opportunistic agents lead to variate CSF patterns Bloods: lymphopenia and thrombocytopenia are frequent in the acute phase of HIV infection (days to weeks after exposure)

Cholera was not included in this table as its neurological manifestations are related solely to metabolic disturbances and no structural changes in the brain imaging or CSF are described

*CT* computed tomography, *MRI* magnetic resonance imaging, *CSF* cerebrospinal fluid, *AFP* acute flaccid paralysis, *T2WI* T2 weighted images, *FLAIR *fluid‐attenuated inversion recovery images, *DWI* diffusion‐weighted images, *SW*I susceptibility weighted images, *AST *aspartate aminotransferase, *ALT *alanine aminotransferase, *CRP *C reactive protein, *LDH* lactate dehydrogenase, *vCJD* variant Creutzfeldt–Jakob disease, *sCJD* sporadic Creutzfeldt–Jakob disease, *ANE * acute necrotising encephalitis, *GBS* Guillain–Barré syndromes, *ADEM* acute disseminated encephalomyelitis, *Sars* severe acute respiratory syndrome, *MERS* Middle East respiratory syndrome, *COVID19* coronavirus disease 2019, *PRES* posterior reversible encephalopathy syndrome, *CNS* central nervous system, *HIV* human immunodeficiency virus, *AIDS* acute immunodeficiency syndrome, *PML* progressive multifocal leukoencephalopathy, *MS* multiple sclerosis, *MIBE* measles inclusion-body encephalitis, *SSPE *subacute sclerosing panencephalitis

### Vector-borne

#### Bubonic plague

The first Plague pandemic, known as the Plague of Justinian, occurred during the sixth century and is believed to have hastened the end of the Roman Empire. The second commenced with a wave known as the “Black Death” and is estimated to have resulted in the deaths of over 100 million people; the highest of any pandemic in history. Currently, most human cases are located in Africa, and annual epidemics are reported in Madagascar [156]. Meningitis is reported in up to 7% of all patients, typically after the 1st week in those who received incomplete or delayed treatment for the bubonic form. Younger patients have higher incidence of Yersinia meningitis (around 11% of children) [8, 14, 82]. It presents as a bacterial meningitis, with neutrophilic pleocytosis in the CSF. The gold standard for diagnosis is the culture of *Y. pestis* in blood, sputum, bubo aspirate or CSF, but the bacteria can also be detected using point of care testing with immunochromatographic assays or quantitative PCR in portable thermocyclers [35, 158].

#### West Nile virus (WNV)

This virus was first identified in the West Nile province of Uganda in 1937 [122]. In 1999 an outbreak occurred in New York which spread to the rest of the Americas, leading to the largest epidemics of meningitis or encephalitis ever reported in the western hemisphere [69]. Under 1% of infected individuals developed the neuroinvasive disease, but the incidence is higher among those > 65 years, which also have higher mortality. The neurologic syndromes can overlap and be divided in meningitis, meningoencephalitis, and acute flaccid paralysis secondary to anterior myelitis and typically occurs in the acute phase of the disease. Extrapyramidal signs are seen in some encephalitic cases during the acute illness, and may be transient or last months after the resolution of the disease. Seizures and encephalopathy have also been reported [123].

#### Japanese encephalitis

Large epidemics of Japanese encephalitis Virus (JEV) occur every 2–15 years in South-East Asia and Western Pacific, making it the world’s most commonly diagnosed epidemic encephalitis (~ 70,000 cases/year). Among the arthropod-borne viruses, JEV leads to the greatest loss of disability-adjusted life years, due to the frequent neurological sequelae of the condition. While the majority of infections are asymptomatic or mild, 0.1–1% cause encephalitis, mainly in children. It manifests in the acute phase of the disease. Symptoms include impaired consciousness, headache, vomiting, and seizures. Pyramidal and extrapyramidal signs, involvement of cranial nerves, eye movement abnormalities and anterior myelitis are described. Similar neurological features are seen in adults. Other immunological manifestations include GBS, ADEM, transverse myelitis and *N*-methyl-d-aspartate (NMDA) receptor encephalitis, weeks after the onset of the viral illness. Approximately one-fifth of patients with JEV-encephalitis die and 44% of those with neurological involvement have incomplete recovery [140].

#### Zika

The Zika virus was described in Uganda in 1947 and has caused outbreaks in Asia and the Pacific. In 2014–2016 it caused an epidemic of microcephaly among newborns in the northeast of Brazil [31]. The congenital Zika syndrome occurs in 5–14% of pregnancies of infected mothers, and includes severe microcephaly, skull collapse, subcortical calcifications, corpus callosum anomalies, decreased white matter, ventriculomegaly, cerebral cortex thinning, abnormal gyral patterns, vermis hypoplasia, chorioretinal atrophy, focal pigmented mottling of the retina, optic nerve atrophy and congenital contractures [90, 94]. Zika virus-associated GBS (both axonal and demyelinating) has been described 5–15 days after the acute disease [94].

#### Chikungunya fever

Chikungunya virus was isolated during an outbreak in 1952 in Tanzania, and significant epidemics have been described worldwide. The most recent started in 2013, affecting the Southern USA, Mexico, Central and South America, with over 2 million infections reported [146]. Young children and older adults are at a higher risk of complications, which may affect approximately one percent of infected individuals. Encephalopathy and encephalitis are caused by direct viral invasion and manifest early during the infection [19]. Myelitis can occur either in the acute phase or later in the course of the disease. Tardive post-infectious complications, thought to be autoimmune in nature, include ADEM, optic neuropathy, GBS, brainstem encephalitis and Bickerstaff’s encephalitis-Miller-Fisher overlap [19].

### Water or food-borne

#### Enteroviruses

Enteroviruses cause over 90% of viral meningitis in children under 10 years and is most frequently by echoviruses and Coxsackie-B [77]. Two enteroviruses that caused epidemics and remarkable neurological manifestations are detailed below.

#### Poliovirus

Poliomyelitis was sporadically reported until the end of the nineteenth century, when large summer epidemics in North America and Europe began occurring annually [96]. The development and implementation of vaccination programmes since 1955 decreased its incidence dramatically, though outbreaks still occur in Africa and Asia, and it is endemic in Nigeria, Afghanistan and Pakistan. Around 1 in 150 infections will lead to paralytic poliomyelitis [96]. When the virus reaches the CNS, there is a meningitic phase followed by spinal poliomyelitis and onset of an acute flaccid paralysis secondary to anterior myelitis, early in the course of the infection. After a period of stable neurological function ($\ge$ 15 years), 30–40% of polio survivors develop progressive and persistent new muscle weakness and increased fatigue, characterising the post-polio syndrome. The pathogenesis is not completely understood but thought to be related to a disturbance of the denervation/re-innervation equilibrium with further denervation. In 2016, it was estimated that there are 15–20 million polio survivors worldwide [76].

#### Enterovirus-71(EV71)

This virus was isolated in 1969 and is a common cause of hand, foot, and mouth disease in children. Cyclical large epidemics occur in the Asian-Pacific region every 2–3 years and it circulates at a low level in the rest of the world, with small outbreaks in Europe, North America and Africa [130]. Children may develop CNS manifestations after 3–5 days of prodrome [100]. During an outbreak in Malaysia, 10–30% of hospitalised children had neurological manifestations, which included aseptic meningitis, encephalitis and acute flaccid paralysis secondary to anterior myelitis, GBS and transverse myelitis [59]. The most common CNS manifestation is brainstem encephalitis, severely affecting the medulla and frequently evolving to cardiac dysfunction and neurogenic pulmonary oedema. Seizures may occur in children under two years. Myoclonic jerks are frequent in encephalitic cases. Up to one-fifth of children with severe neurological manifestations have sequelae, and only a quarter of those with brainstem encephalitis and cardiorespiratory failure have a full neurological recovery [20].

#### Variant Creutzfeldt-Jakob (vCJD)

Bovine spongiform encephalopathy (BSE) was first described in 1985 and peaked in 1992/1993. The first cases of vCJD in humans were described in 1995 in the UK [149], and were found to be caused by the BSE prion [26]. vCJD affects a younger age group when compared to sporadic CJD with a median onset age of 26 years in the UK and 36 years in France [15]. The incubation period may be as long as 15–20 years [27]. In the earliest phase of vCJD psychiatric features are prominent with depression, short-lived delusions and hallucinations being most common [15]. Over 60% of patients may have persistent painful sensory features which are frequently lateralised. The majority of patients present with cerebellar features 4 to 6 months after disease onset. Myoclonus is a late feature, occurring more 6 months after onset, and chorea, tremor and dystonia are also common at this stage. Oculomotor problems and complaints of diplopia may be present in half of patients [15]. All patients develop cognitive impairment (with initial symptoms being disorientation and poor memory) and eventually dementia. Progression to death occurs on average 14 months after disease onset.

### Direct or indirect contact with infected individuals

#### Influenza

Influenza viruses can be divided in seasonal and pandemic. The seasonal influenza A viruses (H3N2 and H1N1) cause yearly epidemics, while pandemics of influenza are the consequence of cross-species transmission, followed by adaption to humans [77]. The CNS is the most common site of extra-respiratory complication of influenza infections [77]. Febrile seizures and encephalopathy are the most frequent neurological manifestations, affecting predominantly children [137]. Other acute neurologic presentations include meningitis, encephalitis (including acute necrotising encephalopathy and acute haemorrhagic leukoencephalopathy), and an increased frequency of ischaemic stroke, all during the acute disease [11, 137]. Influenza may also be associated with Reye’s syndrome, an acute encephalopathy with mitochondrial dysfunction and hepatic metabolic failure, triggered by drugs (especially aspirin) [121].

Late post infectious neurological complications of influenza have been extensively reported and are more frequent in adults. These include including GBS, cerebellitis, Kleine-Levin syndrome, myositis and transverse myelitis [137, 150]. A link between encephalitis lethargica (von Economo disease) and influenza A has been suggested with an outbreak of encephalitis lethargica cases noted after the 1918 Influenza pandemic [56]. During the acute phase, patients presented with excessive sleepiness, disorders of ocular motility, fever and movement disorders; frequently preceded by flu-like symptoms. The chronic phase typically developed 1–5 years after acute disease, but has been delayed by up to 45 years. Symptoms include Parkinsonism with psychiatric symptoms, abnormal ocular movements, speech abnormalities, spasticity and brisk reflexes; a constellation of symptoms memorably described by the neurologist Oliver Sacks in his book “Awakenings” [114]. There remains controversy whether encephalitis lethargica is caused by direct CNS invasion by the influenza virus, or represents a virus-related autoimmune phenomenon [56]. Other cases of post-encephalitic Parkinsonism not related to encephalitis lethargica have been reported after influenza infections [61].

#### Coronaviruses

Since the beginning of the twenty-first century three coronaviruses (CoV) have been responsible for pandemics; severe acute respiratory syndrome (SARS-CoV1) in 2003, Middle East respiratory syndrome (MERS-CoV) in 2012 and SARS-CoV2 (also known as COVID-19) in 2019. Most human coronaviruses cause only mild respiratory symptoms and four strains are endemic worldwide, responsible for up to one third of upper respiratory tract infections in immunocompetent individuals: HCoV-229E, -OC43, -NL63 and -HKU1 [32].

Coronaviruses can invade the CNS and have been associated with many neurological sequelae including demyelinating diseases [4], optic neuritis [33], and Parkinson disease [37]. HCov-OC43 was linked to a case of ADEM [160], fatal encephalitis in an immunodeficient child [91], and to a subset of Chinese children with encephalitis [75].

#### Sars-CoV1

The 2002–2003 pandemic affected > 8000 people in 30 countries, 10% of whom died [71]. SARS-Cov1 was found in CSF samples and brain tissue of encephalopathic patients with symptoms including seizures [72] and optic neuritis, manifesting in the acute phase [157]. Large artery ischaemic stroke were reported in 2.4% [141]. Neuromuscular disorders including myopathy and axonal motor neuropathy were reported among critically ill patients, later during the course of the disease. However, it is not clear whether this is a consequence of direct viral CNS infection, the host inflammatory response, and/or immunologic processes [139].

#### MERS-CoV

MERS has been an ongoing pandemic since initial reports in 2012 and has already affected > 2500 people, 35% of which died [155]. In Saudi Arabia, seizures were reported during the acute phase, in > 8% of patients and confusion in > 25% [113]. ADEM, encephalitis and stroke (possibly due to vasculitis) have also been described [3]. Notably none of these conditions had MERS-CoV detected in CSF. A Korean study reported Bickerstaff’s brainstem encephalitis and polyneuropathy among patients with MERS in the first few weeks of infection [64].

#### SARS-CoV-2

In December 2019, a new coronavirus appeared in Wuhan, China. A large current worldwide pandemic has resulted in six million recorded cases and over 1 million deaths, as of end of September 2020 [62]. Neurological complications of the disease have been reported and its mechanisms are still being scrutinised by the scientific community [36]. A retrospective Chinese study with 214 hospitalised patients described neurological features in just over one third of cohort, including dizziness, headaches and impaired consciousness [83]. A French cohort of 58 critically ill patients reported encephalopathy and corticospinal signs. Brain MRI was performed in 13 patients, 8 of whom had leptomeningeal enhancement and 2 had acute ischaemic lesions [55]. There are reports of COVID patients with rhombencephalitis [151] and meningoencephalitis [159], some with positive Sars-CoV2 in the CSF [92], most of which presented with seizures and encephalopathy. Acute haemorrhagic leukoencephalitis [108], demyelinating lesions [162], ADEM [102] and acute myelitis have also been reported [2]. Peripheral nervous system manifestations include anosmia/ageusia in over 80% of infected [73], GBS (both demyelinating and axonal) [138], Miller-Fisher syndrome (MFS) and isolated abducens palsies [48]. An increased frequency of acute cerebrovascular events among COVID patients is reported [101], at a similar frequency to previous studies of patients with sepsis [12]. This may be the consequence of a hypercoagulable state [164] related either to the viral infection or to the host response [97].

There are increasing reports of many patients suffering from a long-term syndrome lasting more than 3 months post infection which has been badged as “long COVID.” Neurological-type symptoms including neurocognitive difficulties, depression and other mental health conditions, peripheral neuropathies and muscular weakness [46]. This is distinct from critical illness acquired weakness, and the neurocognitive sequelae described in post intensive care syndrome [9], as the majority of the patients reporting this syndrome have not been hospitalised.

#### Ebola

First described in 1976, Ebola has caused several outbreaks, mainly in African countries, the largest in 2014–2016. Neurological complications begin in the late stage when patients can have encephalopathy, seizures (probably due to metabolic abnormalities), meningitis and meningoencephalitis [21]. The exact prevalence of neurological complications in the acute phase is unknown. The CNS may be a reservoir for Ebola virus; it was recovered from the CSF (at higher levels than the blood) of an Ebola survivor 9 months after the patient’s recovery, when it then developed meningoencephalitis and radiculitis [60]. Long-term neurological sequelae are not uncommon among survivors, with memory loss in up to 40%, headaches in one third and muscle pain in 13% [128].

#### Measles

Until the introduction of attenuated measles vaccine, the disease killed 2–3 million people/year [154]. The mortality associated with measles decreased steadily since widespread vaccination programs were put in place in the beginning of the twenty-first century. However, since 2016, declining vaccination rates have resulted in epidemics in all WHO regions, including in previously measles-eradicated areas, like USA and Western Europe [104]. Though pneumonia is the main cause of death, severe CNS manifestations may occur. Primary measles encephalitis (PME) manifests during the exanthem due to direct CNS invasion with seizures, disturbances of consciousness and focal signs. Up to 15% of such patients die and a quarter have permanent neurological damage [16]. The most frequent CNS complication of measles is acute post measles encephalitis, which occurs 2–30 days after infection and affects around 0.1% of children after a measles infection. Another complication is measles-induced ADEM, which begins weeks or months after rash clearance [16, 47]. Prognosis is better than with PME. Measles inclusion body encephalitis (MIBE) is another complication, in which a progressive measles virus brain infection affecting patients with impaired cellular immunity, manifesting within 1 year of the primary infection. It presents as altered consciousness, refractory seizures and focal signs. Mortality is 75% [16, 47]. Subacute sclerosing panencephalitis (SSPE) manifests 4–15 years after an acute measles infection, with a higher incidence in children who had the disease before the age of 5 [47]. SSPE is caused by persistence of a mutant measles virus after failure to completely clear the primary infection, and manifests initially as behavioural changes and cognitive decline, followed by myoclonic jerks, dyskinesias and ataxia, progressing to coma and death [16, 47]. Measles vaccination reduces SSPE incidence.

#### HIV/AIDS

Since the beginning of the pandemic in the 1980s, 75 million people have been infected with HIV, and 32 million have died [152]. While the introduction of combined antiretroviral therapies (cART) has decreased mortality and morbidity of acquired immunodeficiency syndrome (AIDS) and opportunistic infections (OI), the prevalence of complications associated with long-term HIV infections and its treatment have increased, particularly the neurologic ones [95]. Acute HIV infection can cause headache and neck stiffness secondary to aseptic meningitis [119].

The most common CNS OI are tuberculous meningoencephalitis, neurotoxoplasmosis, cryptococcal meningitis, cytomegalovirosis and progressive multifocal leukoencephalopathy (PML) secondary to JC virus [95]. Others include primary CNS lymphoma (associated with Epstein-Barr virus) and varicella-zoster vasculitis, with encephalopathy, cranial nerves palsies, strokes and seizures [119].

Immune reconstitution inflammatory syndrome (IRIS) may occur weeks to months after recovery from an immunodeficient state. Low CD4 before initiation of cART is the strongest predictor of IRIS. It can affect any organ and CNS-IRIS prevalence is around 1%, occurring in response to dying opportunistic agents (frequently linked to Cryptococcus or PML) or as a fulminant encephalitis associated with CD8 + T cells infiltration [95].

Up to 50% of HIV patients may be affected by HIV-associated neurocognitive disorders (HAND) which range from asymptomatic to dementia [52]. HAND is a subcortical cognitive disorder and presents with psychomotor retardation, executive dysfunction, deficits of working memory, retrieval, judgment, attention and impulse control, manifesting as a long-term complication of the infection. In the cART-era the incidence of HIV-related dementia has decreased to under 5% [95], HIV disease markers are no longer closely related to cognitive impairment [50], and patients receiving cART have a better cognitive performance than patients who are cART-naïve [52]. This indicates that the pathophysiology of cognitive impairment may be related to the inflammatory process which occurs in the presence of the virus in the CNS.

HIV infection can lead to a distal symmetric polyneuropathy, which can be related both to neurotoxic antiretrovirals and to the viral infection per se, affecting small fibres and causing numbness and painful distal limbs symptoms. Polyneuropathy affects 30–70% of HIV patients and immunosuppression no longer predicts its severity [103]. It may be related to neurotoxicity secondary to viral replication or to an immune reconstitution mechanism, damaging peripheral nerves and usually manifests in the chronic phase of the disease [95]. GBS (mainly demyelinating) has been associated with HIV very early in the infection [54].

HIV is independently associated with increased risk for stroke which may be secondary to viral effects on endothelial dysfunction, vasculopathy and hyperviscosity [103]. Protease inhibitors used as antiretrovirals may also have a negative effect on vascular endothelial function.

Vacuolar myelopathy manifests in chronic AIDS [119]. An amyotrophic lateral sclerosis-like syndrome has been reported, and may resolve resolved after initiation of cART [95, 119]. Myopathy can occur regardless of the course of HIV infection, and is associated with direct virus lesion, inflammatory response, or ART (zidovudine) [95].

## Other diseases and potential threats

Increases in global temperatures and a changing climate can lead to environmental adaptations of benefit for various disease vectors, including mosquitoes. These are the key vector for malaria, whose epidemic potential should increase in susceptible tropical countries (extending to highland areas) that had controlled the disease or be reintroduced in temperate climates that had previously eliminated it. The most severe form of malaria (and cause of 500,000 deaths per year) is cerebral [107], affecting mainly young children during the acute illness.

Yellow fever, another mosquito-borne virus, was a major threat to human health until the beginning of the twentieth century, having caused multiple epidemics and deaths in cities distant from endemic areas, in North America, the Caribbean and Europe [89]. The expansion of the disease to non-endemic areas means that susceptible non-vaccinated populations are now prone to new epidemics. The virus can rarely invade the CNS and cause encephalitis and meningitis early in the course of infection. Encephalopathy is also common in the context of hepatic failure of severe forms of the disease. Though extremely rare, yellow fever vaccine-associated neurotropic disease is reported, causing encephalitis, GBS and ADEM [89].

The mosquito-borne dengue virus has expanded from a sporadic disease affecting 9 countries in the 1970s to being endemic in over 100 countries; half of the world is now at risk. Neurological complications can occur at any stage. Dengue encephalopathy is the most common and involves impaired consciousness in the context of shock, liver failure and electrolyte disturbances in the first 10 days of the disease. Meningitis and encephalitis due to CNS invasion of the virus are also possible, though rare, and patients present with decreased level of consciousness, headache, dizziness, seizures and focal signs, also in the acute phase. Post-dengue immune-mediated complications include GBS, transverse myelitis, ADEM, mononeuropathies of cranial nerves, optic neuropathy, muscle dysfunction and intracranial haemorrhages during the convalescence stage [17].

War, conflicts and natural disasters can facilitate the spread of diseases like cholera. Cholera has caused seven pandemics in the last two centuries. Electrolyte disturbances and hypoglycaemia (mainly in children) secondary to severe diarrhoea and acute dehydration can lead to symptomatic seizures [23]. Concurrent outbreaks of cholera and peripheral neuropathies have been described among undernourished displaced populations, in the subacute phase of the disease [112].

The recent decrease in coverage of MMR (measles, mumps and rubella) vaccine in some areas has also led to an increase of cases of rubella and mumps. Mumps can cause aseptic meningitis and encephalitis in the early days of the disease and ADEM as a late post infectious complication [6]. The major neurological manifestation of rubella is the congenital rubella syndrome (CRS) in foetuses whose mothers are infected during pregnancy. CRS includes causes encephalopathy, microcephaly and sensorineural hearing loss. Encephalitis has also been reported during the exantematic phase [6].

Smallpox was a cause of massive epidemics until its eradication in the 1980s by global immunisation programs. Since most of people living today are not vaccinated against it and the viable variola virus is still kept in two maximum security laboratories [87], it is feared that it could be used in bioterrorist attacks. Neurological complications of smallpox were uncommon and have been poorly studied, but descriptions are compatible with demyelinating/inflammatory encephalomyelitis, 5 to 16 days after the acute disease [13]. Post-vaccination encephalomyelitis has also been described, mainly in young children [13].

## Conclusion: preparing for the future

Pandemics and epidemics have been present for thousands of years, and have played a pivotal role in history. Much of previous focus has been on the acute illnesses themselves, with relatively little attention paid to the social, human and economic consequences of neurological sequelae. However, it is these sequelae that often lead to significant amounts of mortality and long-term morbidity.

Under-recognition of neurological manifestations means that few studies have been conducted in previous pandemics to understand, treat and prevent neurological complications, and so the burden of secondary complications is even greater. With the recent developments in imaging, new information about the presentation of CNS diseases is available and can assist in the proper diagnosis of neurologic manifestations of infectious diseases. However, even in developed countries, diagnostic tests are limited and treatments are often inadequate or non-existent, with significant long-term economic and healthcare consequences. Therefore, it is reasonable to expect that in low and middle-income countries with poorer access to diagnostic tests and treatments, the neurological involvement by these diseases will have a greater economic impact. Potentially limiting future human capital by leaving long-term motor and cognitive impairments.

This urgent need to pay more attention to the short- and longer term sequelae of pandemics has been brought into sharp focus with COVID-19 where there have been numerous reports of short-term sequelae and a growing appreciation of longer term problems. There is a growing recognition of the need to work globally with international collaborations being formed to better understand the neurological consequences of COVID-19 [53]. These include (but are not exclusive to) the CoroNerve Study Group [143], European Academy of Neurology’s EAN Neuro-COVID Registry Consortium (ENERGY) [98], and the Global Consortium Study of Neurological Dysfunction in COVID-19 (GCS-NeuroCOVID) [40].

However, it is also important to learn from previous pandemics to understand what to expect and plan responses to improve the outcomes. This knowledge of what has occurred the past is useful to highlight symptoms and signs to be vigilant for ensuring such sequelae are not missed. For example, encephalitis lethargica and post-encephalitic parkinsonism have been seen after viral infections, most notably the 1918–19 influenza pandemic [43]. Indeed, a case of parkinsonism in a patient post COVID-19 has been described, including a reduction of ^18^F-fluorodopa uptake bilaterally in the putamina [25], In the months to years following this pandemic it would be important to continue to look for such patients.

As new infections continue to emerge, new pandemics will certainly happen. Despite advances in the treatment of infectious diseases in the last century, those affecting the nervous system are still challenging. An improved understanding of the pathophysiology of neurologic damage and recognising its possible manifestations is fundamental to develop new treatments and management strategies.

Neurological involvement in pandemics and epidemics is common and can cause devastating consequences amongst affected populations. There is an urgent need for better address this issue in pandemics, including the current SARS-CoV-2 outbreak.
